# Robust Orientation Estimation from MEMS Magnetic, Angular Rate, and Gravity (MARG) Modules for Human–Computer Interaction

**DOI:** 10.3390/mi15040553

**Published:** 2024-04-21

**Authors:** Pontakorn Sonchan, Neeranut Ratchatanantakit, Nonnarit O-Larnnithipong, Malek Adjouadi, Armando Barreto

**Affiliations:** Electrical and Computer Engineering Department, Florida International University, Miami, FL 33174, USA

**Keywords:** MEMS MARG orientation, orientation for human–computer interaction, magnetic distortion, GMVDμK algorithm, GMVDK algorithm

## Abstract

While the availability of low-cost micro electro-mechanical systems (MEMS) accelerometers, gyroscopes, and magnetometers initially seemed to promise the possibility of using them to easily track the position and orientation of virtually any object that they could be attached to, this promise has not yet been fulfilled. Navigation-grade accelerometers and gyroscopes have long been the basis for tracking ships and aircraft, but the signals from low-cost MEMS accelerometers and gyroscopes are still orders of magnitude poorer in quality (e.g., bias stability). Therefore, the applications of MEMS inertial measurement units (IMUs), containing tri-axial accelerometers and gyroscopes, are currently not as extensive as they were expected to be. Even the addition of MEMS tri-axial magnetometers, to conform magnetic, angular rate, and gravity (MARG) sensor modules, has not fully overcome the challenges involved in using these modules for long-term orientation estimation, which would be of great benefit for the tracking of human–computer hand-held controllers or tracking of Internet-Of-Things (IoT) devices. Here, we present an algorithm, GMVDμK (or simply GMVDK), that aims at taking full advantage of all the signals available from a MARG module to robustly estimate its orientation, while preventing damaging overcorrections, within the context of a human–computer interaction application. Through experimental comparison, we show that GMVDK is more robust to magnetic disturbances than three other MARG orientation estimation algorithms in representative trials.

## 1. Introduction

Lee et al. [1] propose that “The first micromachined accelerometer appeared in 1979 at Stanford University”, referring to the device published by Roylance and Angell [2]. However, Lee et al. also point-out that “it took over 15 years before such devices became accepted mainstream products for large volume applications. In the late 1980s, surface micromachining emerged as a perceived low-cost alternative for accelerometers, aimed primarily at automotive applications” [1]. It would not be until 2009 that the motion sensing capabilities of micro electro-mechanical systems (MEMS) would be supplemented with commercial three-axis gyroscope chips [3]. Soon, the combination of a tri-axial accelerometer and a tri-axial gyroscope would be made available into packages designated as “MEMS inertial measurement units” (MEMS IMUs), which prompted the expectation that, perhaps, it would be possible to use them to track the position and orientation of objects in ways similar to the methods used to track ships and aircraft from signals generated by full-size, navigation-grade accelerometers and gyroscopes usually present in those vehicles. Those large vehicles are commonly tracked using the principles of “strapdown inertial navigation” [4,5,6].

Unfortunately, the past two decades have shown that the traditional signal processing approaches that are successful in tracking vehicles from navigation-grade accelerometers and gyroscopes could not merely be re-adopted when the signals are being produced by MEMS IMUs, as detailed in the next section.

### 1.1. Need for a Different Approach

The mere re-adoption of algorithms used to track large-scale vehicles is not feasible because the performance indices of low-cost MEMS accelerometers and gyroscopes are significantly below those of their full-size, navigation-, or tactical-grade counterparts. This is, essentially, the conclusion reached by Foxlin [7] after studying the motion tracking requirements for virtual environments. Foxlin clearly identifies one of the major shortcomings of MEMS IMUs: “The problem with tracking orientation using only gyros is drift”, where drift is the gradually increasing error in the orientation estimate. Further, Foxlin reveals the gyroscope bias (non-zero gyroscope output when no rotation is taking place) and the gyroscope bias instability (shift in the amount of bias as time progresses) as important causes for the orientation drift error, pointing out that “Bias stability is often the critical parameter for orientation drift performance”. Table 1 shows the values (extracted from Figure 8.4 in [7]) of Gyro bias stability and Gyro bias initial uncertainty that Foxlin presents as typical of the different categories (grades) of IMUs.

It is noticeable in Table 1 how the critical parameters of “commercial-grade” gyroscopes (such as miniature MEMS gyroscopes) have values that are orders of magnitude worse than those of strategic, navigation, or even tactical IMUs. This lower performance of MEMS IMUs prevents their direct use in the “strapdown” configuration previously employed to estimate both the position and the orientation of a rigid body, with respect to an external, immobile “reference” or “inertial” frame of coordinates. In the strapdown approach, the accelerometer and gyroscope of the IMU are affixed to the moving object, i.e., to its “body frame”. Every time the gyroscope measurements are read, they are added to a running tally that indicates, therefore, the total rotation (orientation change) of the object from its initial orientation. That is, in this “dead reckoning” approach, the integration of gyroscope signals estimates the current orientation of the “body frame” with respect to its initial orientation in the “inertial frame”. The accuracy of this first estimate of orientation is paramount because it is relied upon to project or “resolve” the readings from the accelerometer (along the directions of the “body frame”) to the axes of the inertial frame where the known acceleration of gravity is subtracted from them, to leave only the so-called “linear” acceleration components (in the inertial frame). If the intended operation of the system were correct until this point, each of the linear acceleration components could then be “double-integrated” (in ways similar to the integration of gyroscope readings) to yield the displacement components along the inertial axes, i.e., the position of the moving object from its initial location, in terms of the inertial frame. The stringent demand for gyroscope accuracy in this scheme is underscored by the realization that any error in the orientation estimated from the gyroscope will lead to an inaccurate resolution of accelerations to the inertial frame, which will introduce errors in the inertial frame accelerations upon the incorrect removal of gravity. Through the necessary double integration of accelerations, the resulting position errors will grow as a quadratic function of time. Accordingly, Foxlin states that, for this approach, “An even more critical cause of error in position measurement is error in the orientation determined by the gyros” [7]. Based on simulations of strapdown configurations with accelerometers and gyroscopes of different grades (commercial, navigation, tactical, strategic), he concluded, referring to the use of MEMS IMUs, that “Therefore, human-motion tracking systems that can maintain position to a few centimeters for more than a minute without external correction are not on the horizon.”

Despite improvements in MEMS accelerometers and gyroscopes made in the years after Foxlin’s simulation, their successful operation in a strapdown configuration continued to be unfeasible. Woodman, in his 2007 report concluded “It is not currently possible to construct an INS which maintains sub-meter accuracy for more than 60 s using MEMS devices” [8].

The bias level and variability of commercial-grade MEMS gyroscopes are typically higher in comparison to other types of gyroscopes. For example, a white paper published in 2014 by KVH Industries, Inc. (Middletown, RI, USA) [9], a manufacturer of high-end inertial systems, characterizes the “bias offset” of MEMS gyroscopes (at 25 °C) as “± 250°/h” with “bias instability (at constant temperature)” of “less than 1°/h, 1σ”, as compared to only “±2°/h” and “less than 0.05°/h, 1σ”, respectively, for fiber optic gyros (FOGs), which are more expensive, larger, tactical-grade gyroscopes.

Technological advances in recent years have achieved important reductions in the offset level and instability of some MEMS gyroscopes, although the most significant improvements are mainly found in research devices or specialized commercial devices with costs that would be prohibitive for instrumenting a computer interaction glove with 10 or more of them. For example, Wu et al. published their development of a “sub-0.1°/h bias-instability MEMS gyroscope” in 2021 [10]. In their device, they pursue the strategy of “resonant constant frequency” (RCF) to ensure “that the excitation frequency and resonant frequency are equal and constant” in order to “eliminate effects of excitation-frequency instability and drift on gyroscope output performance.” More specifically, this same group combined RCF with real-time automatic mode-matching, achieving a measurement of “bias instability of 0.09°/h” in an experimental device [11]. More recently, Bu et al. [12] proposed an “online compensation method for ZRO drift based on multiparameter fusion”. Upon application of their approach, they observed that “after online compensation, the BI [bias instability] reached 0.23°/h”. This demonstrates that technological improvements can yield much smaller offset levels and instability, but sophisticated approaches are mainly commercially available in high-cost MEMS gyros, such as the Tronics/TDK (Crolles, France) GYPRO3300 one-axis (yaw) MEMS Gyroscope, which retails for more than USD 600 per unit. According to its datasheet [13], this device has a bias instability specified as typical = 0.8°/h, maximum = 3°/h. On the other end of the cost spectrum for commercial MEMS chips that integrate three-axial gyroscope, tri-axial accelerometers (and possibly tri-axial magnetometers), such as the Bosch (Gerlingen, Germany) BMI088 MEMS 6 DOF IMU (retailing for less than USD 10 per unit), continue to have large values bias instability (e.g., the “zero-rate offset” is specified as “± 1°/s” in the 2024 datasheet for the Bosch BMI088 MEMS IMU [14]). Our focus throughout this article is on MEMS MARG modules in the cost range that would still make it affordable to instrument an interaction glove with 10 or more of the devices. In particular, we experimented with the 3-Space™ Micro USB MARG module from Yost Labs (Portsmouth, OH, USA) specified with a “Gyro bias stability @ 25 °C 2.5°/h average for all axes”. In addition to the relative affordability of these modules, we selected them for our experimentation due to their ease of connection to a computer (via a USB cable) and the availability of the C#-language-based application programming interface (API) that facilitated the software development in our experiments. Importantly, the API is directly compatible for the use of the “Nano” version of the 3-Space MARG (retailing for USD 39 per unit). The complete set of specifications offered by the manufacturer is transcribed in Appendix A.

In view of the difficulties for obtaining reliable tracking estimates from just gyroscope and accelerometer readings, some manufacturers also included a tri-axial magnetometer in packages advertised as “9-degrees-of-freedom IMUs” or magnetic and inertial measurement units (MIMUs)”, or, the designation we prefer, “magnetic, angular rate, and gravity (MARG)” sensor modules. One of the earliest of these MEMS sensors modules was InvenSense’s MPU-9150, initially released in 2011 [15].

This paper presents our algorithm to obtain orientation estimations for a low-cost MEMS MARG module in real time, guarding against the estimate degradation that could occur in environments with large ferromagnetic objects, for the purpose of human–computer interaction.

### 1.2. Prediction–Correction of MARG Orientation

The unavoidable presence of time varying bias in the signals from the gyroscopes contained in low-cost, miniature MEMS MARG modules, along with the single and double integrations required as part of the strapdown approach, have led to re-focusing the goal of MARG tracking to estimation of the orientation of the module alone (which, in any case, would be a prerequisite to the implementation of the strapdown approach for position tracking).

This has prompted attempts to implement classical methods for orientation estimation that combine an initial estimation generated from gyroscope data, followed by a correction phase frequently achieved with the involvement of a different sensor modality, for example, accelerometer data, and also potentially involving magnetometer readings.

One of the most popular prediction–correction algorithms used in the navigation arena is the Kalman filter [16], which is an optimal linear estimator in the sense that it minimizes the variance of the estimates obtained [17]. However, application of Kalman filtering for orientation tracking of low-cost MEMS MARG modules has resulted in limited success, as we discuss further in Section 1.6.

### 1.3. Scope of Our Approach to Prediction–Correction MARG Orientation for HCI

From the previously presented considerations, we sought to define an alternative way to make use of the three types of signals available from a low-cost MEMS MARG module in the context of a hand-tracking application for human–computer interaction. This means that we would restrict our focus to uses of the commercial-grade, low-cost MARG modules, considering them attached to hand-held human–computer interaction devices, or embedded in an interaction glove, to be worn by the computer user. It should be noted that the same kind of context surrounds the utilization of MEMS MARG modules in many Internet-of-Things (IoT) applications.

Our specific focus on human–computer interaction applications allows us to establish two important expectations for the operation of the MARG:

(a) As we expect the MARG to be affixed, in one way or another, to the hand of a human computer user, we can expect that there will be intervals in which the MARG will be static (or very close to it), occurring frequently.
(b) The overall travel of a MARG in any particular use run will be constrained to the scale of meters. This, in fact, implies that objects around the MARG (and, in particular, large ferromagnetic objects) will remain in static or slowly moving relative positions with respect to the MARG.

(Both these expectations are also plausible in many IoT potential uses of the low-cost MEMS MARG modules.)

### 1.4. New View of the Information Sources Onboard a MARG Module

For application in HCI studies, the focus, then, will be to estimate the orientation of the coordinate frame considered attached to the body of the MARG module (the “body frame”) with respect to an external, immobile frame of reference, which we will call here the “inertial frame”. The extent of the overall movement of a MARG attached to a segment of the human body will be small enough so that a corner of the floor in a room (or the intersection of two perpendicular streets) can be proposed as the origin of the (Cartesian) inertial coordinate frame, with the third axis passing through that corner and following the orientation of a plumb line. To achieve the estimation of the MARG orientation, our algorithm seeks to make comprehensive use of the readings of all three sensor modalities available in the MARG module, but must, simultaneously, scale down the involvement of any given sensor modality when the assumptions made for its utilization are not sufficiently met. Using information from a sensor modality in an unvetted, unbridled fashion may result in effectively adding error to the recurrent estimates of MARG orientation obtained. The assumptions made for each of the three kinds of sensors are explained next.

### 1.5. Estimation of Orientation Differences

Orientation is, in effect, a relative measurement. Much like the measurement of electrical potential (voltage), which is expressed as a difference in potential from a test point to a reference point, we seek to measure the difference of orientation between the MARG body frame and the inertial frame. Therefore, we describe the orientation of the MARG as the rotation that, if applied to the body frame, would take it from an initial state, where it was aligned with the inertial frame (no orientation difference), to its current orientation.

It should be noted that this paper assumes that both the inertial frame and the MARG body frame are Cartesian coordinate systems, adhering to the “left hand rule” (this means that if the left hand is put in a flat configuration, with the fingers initially pointing in the direction of the positive X axis, and then the pinky, ring, middle, and index finger are bent 90 degrees to match the direction of the positive Y axis, the extended thumb will point in the direction of the positive Z axis). It is also important to indicate here that we (as many contemporary researchers in the field) have chosen to express the MARG orientation estimate resulting from our algorithm as a quaternion, which is a representation of 3D rotation [18,19,20]. That is, we identify the estimated MARG orientation with the rotation that would turn the MARG from its initial orientation to its “current orientation”, at any time. Further, we invoke the assumption (without loss of generality; if a known rotation mediates between the desired inertial reference frame and the startup orientation of the MARG, it would just be necessary to “compound” that rotation with the ***q***_OUT_ result from our algorithm after each iteration (see Section 2.1)) that the initial orientation (at “t = 0” or at “startup”) of the “body frame” of the MARG coincides with the fixed orientation of the inertial frame.

#### 1.5.1. Inferring Orientation from Gyroscope Signals

The tri-axial gyroscope in a MARG reports the angular speed at which the MARG has rotated (with respect to the X, Y, and Z axes of its body frame) in the last interval of observation, which we denote as ΔT. Under the assumption that the rotations (in the three body axes) took place at an approximately constant angular speed during the interval of observation, the change of orientation completed during the interval of observation is the product of the angular speed multiplied by ΔT. Tracking systems keep a running tally of the total orientation change over multiple observation intervals by accumulating the newest orientation change to the previous total that had been reached prior to the latest observation interval. Then, this tally represents the rotation that would be necessary to take the MARG from its initial orientation (coincident with the orientation of the inertial frame) to its current orientation, i.e., it estimates the orientation of the MARG.

However, we have already mentioned the unavoidable imperfections present in the operation of MEMS gyroscopes, characterized by their bias (non-zero reading obtained when no rotation is taking place), and, most critically, their bias instability, which expresses the magnitude of change in bias that can be expected in a gyroscope, as time progresses. This variability of the gyroscope bias makes it impossible to achieve a single bias compensation (when the sensor is initialized) that would continue to completely eliminate the effective bias for any significant interval of time. More specifically, Aggarwal et al. identify at least two aspects of the bias uncertainty. First, the “run-to-run” gyroscope bias is such that “every time the sensor is switched on, a slightly different bias or scale factor is observed” [21]. Even more critically, the “in-run” gyroscope bias stability represents an “error that occurs due to change in bias or scale factor during a run” [21]. Further, the behavior of the bias and its evolution has been found to be significantly affected by temperature differences. “The temperature-dependent variations can be quite pronounced in very low-cost MEMS sensors” [22].

Given that the mechanism for orientation estimation from gyroscopic measurements unavoidably requires the accumulation, i.e., the integration, of the rates of rotation read from the gyroscope at every sampling interval, any slowly varying uncompensated remnant of bias in the gyroscope will result in the growing orientation error referred to as “drift”. To the best of our knowledge, there are no bias compensation algorithms, currently, which can completely eliminate the bias of low-cost MEMS gyroscopes on a permanent basis. Therefore, the orientation estimates resulting from gyroscope signal integration must be corrected periodically to prevent the drift error from growing to levels that will render the orientation estimations irrelevant.

#### 1.5.2. Inferring Orientation Information from Accelerometer Measurements

Each axis of the tri-axial accelerometer in a MARG really measures forces acting on a known test mass. Therefore, each accelerometer channel (axis) responds to the “linear acceleration” associated with change of speed of the MARG in the corresponding direction but also responds to the component of the gravitational acceleration in that direction. While the “strapdown” inertial measurement systems focused on detecting the total accelerations, resolving them to the inertial frame and then removing the components due to gravity, most contemporary MEMS MARG orientation estimation approaches seek to detect the components of gravity (exclusively) to use it as a “vector observation” [23]. This means that the gravitational acceleration is viewed as a uniform vector field in the space where the MARG is operating. In consequence, the way in which gravitational acceleration measurements along the X, Y, and Z body axes change, from the original orientation at startup (when the body frame and the inertial frame were aligned) to the current orientation of the MARG, must be the result of the rotation that mediates between the two frames. As such, the difference in X, Y, and Z axes readings contain information about the rotation of the MARG from startup to its current orientation. Observation of just one vector field would not be enough to determine the rotation between the inertial frame and the current MARG body frame, as indicated by Shuster and Oh [24]. (Nonetheless, the information obtained from observing even a single vector field may be used to correct a preliminary orientation estimate, as we do in our approach.) However, a critical assumption to extract correcting information from the difference between accelerometer axes now and at startup is that the readings are dominated by the gravitational acceleration. That is, the assumption will only be practically verified when the MARG is static or close to it, such that the non-gravitational “linear acceleration” contributions included in the accelerometer readings are negligeable with respect to the gravitational contributions. During intervals when this assumption is not verified, applying strong orientation corrections based on accelerometer readings may introduce errors in the orientation estimates.

#### 1.5.3. Inferring Orientation Information from Magnetometer Measurements

The inference of orientation information from magnetometer readings follows the same basic rationale as for the accelerometer readings. That is, if the geomagnetic field in the operating space of the MARG were considered uniform, orientation information can be derived from the magnetometer readings as one “vector observation”. Fortunately, the magnetometer readings are not altered if the MARG is moving or static. However, in this case, the magnetic field in the area of operation of the MARG may, itself, be non-uniform in certain regions, with some areas exhibiting permanent distortions. This will happen in the neighborhood to ferromagnetic items, such as furniture parts made of steel or iron in offices, classrooms, and laboratories [25], or even structural elements involved in the construction of most contemporary buildings [26]. Due to their higher magnetic permeability, ferromagnetic objects will provide an “easier” path for lines of magnetic flow. Because of this, the magnetic field vector in the neighborhood of these objects may point in a different direction than it does in the regions not affected by neighboring ferromagnetic objects. The permanent or slow varying distortion of the magnetic field produced by these objects is particularly disrupting to the assumption of a uniform (in magnitude and direction) magnetic field (more so than other rapidly alternating variations of the field). In consequence, the inference of MARG orientation from magnetometer readings also has a precondition for it to be valid (the magnetic field at the current location of the MARG should not be distorted).

### 1.6. Relevant Previous Work

The potential to track the motion of diverse segments of the human body has been highly attractive to several research communities for a long time. The biomechanics research community might have been one of the earliest to try to take advantage of inertial sensors for their work [27,28], among others. Similarly, the human–computer interaction community saw in MEMS MARG modules the possibility to develop an unobtrusive mechanism to achieve real-time monitoring of the motions of a computer user to provide input to computer systems. This was particularly attractive at the time in which the technologies driving virtual reality were emerging.

Unfortunately, the sobering findings of Foxlin [7] and Woodman [8], which we have already mentioned, among others, made it clear that the full the potential of MEMS IMU and MARG modules for human movement tracking would not be immediately realized. In fact, there has been a continuous tug-of-war between those sensor limitations and the enthusiasm for developing promising motion tracking systems with MEMS MARG modules. As an example, there have been continued efforts to develop a system, based on MEMS MARG modules, that could track the movement of the hand in real time. In particular, the group from University of Twente has continued to develop a system for real-time ambulatory assessment of hand kinematics that involves the attachment of multiple MARG modules to each finger [29,30], which were later consolidated in their “Power Glove” [31]. That type of system continues to be pursued by multiple groups, such as the one from National Taipei University, whose glove combines MEMS MARG modules with force sensing resistors (FSRs) for the assessment of hand function [32]. Any of these applications, however, requires the availability of a reliable and robust algorithm to estimate the orientation of each individual MARG used.

Many approaches to achieve the desired robustness in the orientation estimates have been attempted. A systematic and comprehensive review of those approaches was recently carried out by Nazarahari and Rouhani [33]. In that broad review, they identify three major groups of approaches: those based on vector observations (VO); those that are fundamentally a complementary filter (CF); and those that stem from the Kalman filter, as one of the most popular state estimators for the study of dynamic systems. Nazarahari and Rouhani also performed a critical comparison of 36 of the algorithms mentioned in their survey [34], which they organized into seven major groups: the linear (LCF) and nonlinear (NLCF) complementary filters, and five varieties of Kalman filters: linear Kalman filter (LKF); complementary Kalman filter (CKF); extended Kalman filter (EKF); square-root cubature Kalman filter (SRCKF); and square-root unscented Kalman filter (SRUKF).

Indeed, several variants of the Kalman filter have been used for orientation estimation on the basis of inertial measurements since the 1960s [35,36], but they have been less successful when applied to sensors (e.g., MEMS MARG modules) that have lower levels of performance than the aeronautical and space inertial sensors.

In our view, one aspect that may contribute significantly to the detriment of Kalman-based approaches is the covariance matrix representation used in Kalman filters for the uncertainty associated with the “measurements” (e.g., accelerometer and magnetometer readings) involved in the correction phase of the algorithm [17]. The covariance matrix representation of uncertainties makes it difficult to keep those representations updated to exactly reflect the current conditions in which the MARG is operating. If these uncertainty representations are not correct, the Kalman correction phase maybe counterproductive. For example, if magnetometer readings are used for the correction phase of the Kalman filter, with the implicit assumption that the magnetic field is constant in magnitude and direction at all the locations where the MARG will be used, and then the module is used in an area where the magnetic field uniformity assumption is violated, the correction may introduce error in the orientation estimate. Furthermore, since the Kalman filter is a recursive estimator, the injection of error will continue to degrade the orientation estimate for multiple iterations in the future. Some approaches have addressed the correction step of Kalman filters in alternative ways that seek to “alter” the strength of the corrections from accelerometer and magnetometer measurements, factoring instantaneous conditions. One example is the attempted scaling of the measurement covariance matrix, R [37]. These approaches, however, are challenged by the difficulty in modifying each of the multiple (e.g., nineentries in a measurement covariance matrix according to their true meaning (i.e., auto-covariances along the diagonal and cross-covariances elsewhere). Properly estimating the necessary changes to each one of the entries individually is challenging and, commonly, the adjustment is simply implemented as the use of a different scalar factor that multiplies the whole covariance matrix.

A new approach for the correction of an initial orientation estimate that allows for the easier modulation of the influence of the correcting factors has emerged. In this approach, the correction is carried out as an interpolation between the preliminary orientation estimate quaternion and a quaternion representing an orientation that has been corrected with the “full strength” of the information derived from the “measurements” (e.g., the current magnetometer readings). As an example, Valenti et al. explored the use of quaternion interpolation, although they still implemented the concept as auxiliary to a Kalman filter [38]. Further, they find the “full strength” correction quaternions by exclusively algebraic means, as made evident in the name of their approach: the Algebraic Quaternion Algorithm (AQUA).

Our method, which implements the corrections driven by the accelerometer and the magnetometer as quaternion interpolations, was formulated on an intuitive basis, as explained next, and does not involve the structure of a Kalman filter in any way.

## 2. Materials and Methods

In this section, we describe the proposed algorithm for MARG orientation estimation, including the mechanism we are now using to compute of magnetometer trustworthiness, μ_K_, without using any source of information that is external to the MARG module itself.

### 2.1. GMVDK Algorithm for MARG Orientation Estimation

In the development of the original “Gravity and Magnetic North Vector correction -Double SLERP” (GMVD) algorithm, we considered the availability of tri-axial gyroscope, accelerometer, and magnetometer readings from the MARG itself every Δt seconds and a simultaneous reading of X, Y, and Z MARG position. The position readings were provided by a module containing 3 infrared cameras, which tracked an infrared reflective surface applied on the MARG. (The next subsection describes how this last input of information has now been substituted, such that it is no longer necessary, in the new GMVDK algorithm.)

The goal of our algorithm is to generate, after every new batch of sensor data becomes available, a MARG orientation estimation that takes full advantage of all three sensing modalities present in the MARG, but, simultaneously, protects the estimate from being “contaminated” with invalid influences from the accelerometer and magnetometer, when the preconditions for their corrections are not met. GMVD includes very deliberate provisions to reduce the negative impact that the involvement of the magnetometer could have when the MARG is operating in a region of space where the geomagnetic field is distorted.

We chose to represent the current orientation of the MARG as the rotation that would be necessary to re-align the fixed inertial frame with the current body frame of the MARG. To simplify the process, we define the initial MARG orientation (at “startup”) to coincide with the orientation of the inertial frame. (If a different initial MARG orientation is used, the corresponding rotation from the inertial frame would only need to be known and “added” (“compounded”) to the final orientation estimate obtained from each iteration of GMVD). We have chosen to represent the 3D rotation that defines the orientation of the MARG through a unit quaternion (i.e., a quaternion with unit norm). This is a common choice in orientation estimation as this representation is compact, avoids ambiguities such as the “gimbal lock” effect, and is commonly used to drive 3D animation environments (e.g., Unity^®^) for visualization purposes. Therefore, we seek to find a quaternion, ***q_OUT_***, to represent the orientation of the body frame as the rotation that would modify the orientation of the inertial frame to match the current orientation of the body frame.

There are several excellent books that describe quaternions, their manipulations, and their geometrical interpretations [18,19,20]. Here, we only summarize critical aspects of quaternions that are necessary for the explanation of our method. A quaternion, ***q***, is represented as a four-element array of real numbers, such as $q={\left[\;q_{x},\;q_{y},\;q_{z},\;q_{w}\;\right]}^{T}$, where 3 of them (*q_x_*, *q_y_*, *q_z_*) are called the “vector part” and the fourth number, *q_w_*, is called the “scalar part” of the quaternion. We signify quaternions and vectors with boldface. When necessary, we use this operator to form a quaternion from a 3D vector, ***q_v_***, and a scalar *q_w_*:

(1) $$q=H(q_{v}\;,\;q_{w})$$

The addition of quaternions, the product of a scalar times a quaternion, and the inner product between quaternions are the same as the corresponding operations for four-element vectors, but the product of quaternions is defined in a completely different way [18,19,20] and is symbolized as “⊗”. A quaternion norm can be computed as the square root of the sum of all 4 quaternion elements raised to the second power. Any quaternion can be modified to be a “unit quaternion” by calculating the norm of the quaternion and then dividing each of its components by that norm. The conjugate of quaternion ***q*** is simply $q^{*}={\left[-q_{x},\;{-q}_{y},-q_{z},\;q_{w}\right]}^{T}$.

A quaternion of unit norm can be used to represent a 3D rotation, around a 3D vector ***u*** = [*u*_x_, *u*_y_, *u*_z_] (which does not need to be parallel to any of the X, Y, or Z axes of the coordinate frame). In that representation, the amount of rotation around the ***u*** axis is *β*, and the quaternion components will satisfy [39]

(2) $$q_{w}=cos\left(\frac{\beta }{2}\right)$$

(3) $$\left[\begin{array}{c} q_{x}\\ q_{y}\\ q_{z} \end{array}\right]=u\;sin\left(\frac{\beta }{2}\right)=\left[\begin{array}{c} u_{x}\\ u_{y}\\ u_{z} \end{array}\right]\;sin\left(\frac{\beta }{2}\right)$$

(It should be noted, then, that the quaternion ***q***_NOROT_ = [0, 0, 0, 1]^T^ indicates “no rotation”). Frequently, the interplay between quaternions and “normal” 3D vectors, such as the ones one would use to indicate a 3D direction, of, for example, the force applied by a rope pulling a mass, takes place by means of “casting” the 3D vector ***v*** = [*v*_x_, *v*_y_, *v*_z_] as a “pure” quaternion by adding a scalar component with a value of zero: ***q***_v_ = [*v*_x_, *v*_y_, *v*_z_, 0]. Conversely, if a quaternion has 0 scalar component, it can be “re-interpreted” as just a 3D vector.

There are two critical implications of the use of quaternions to represent the 3D rotations we leverage in our method. First, if a unit quaternion ***q*** represents the orientation of the body frame with respect to the inertial frame (i.e., the rotation that would align the inertial frame to the body frame), a 3D vector whose coordinates in the inertial frame are ***v_i_*** = *v*_ix_, *v*_iy_, *v*_iz_ can be “mapped” (from inertial to body frame) with the following quaternion operation to find the coordinates of that same 3D vector in the body frame ***v*_b_** = *v*_bx_, *v*_by_, *v*_bz_. (For this, ***v_i_*** must first be “cast as pure quaternion”, and the initial quaternion result of the equation must finally be “re-interpreted” as a 3D vector.)

(4) $$v_{b}=\;q^{*}\;⊗\;v_{i}\;⊗q=mapItoB(q,v_{i})$$

Because of the practical effect of the above equation, we refer to the process described in it as ***v*_b_** = *mapItoB*(***q***, ***v*_i_**). Similarly, a 3D vector initially defined in the body frame, ***v*_b_**, can be mapped to the inertial frame with this operation, which we refer to as ***v*_i_** = *mapBtoI*(***q***, ***v*_b_**).

(5) $$v_{i}=\;q\;⊗\;v_{b}\;⊗\;q^{*}=mapBtoI(q,v_{b})$$

Second, within a single frame of reference (e.g., the body frame), a 3D vector can be rotated from an initial direction 1, where its coordinates are ***v*_1_** = *v*_1x_, *v*_1y_, *v*_1z_ to a final direction 2, where its coordinates will be ***v*_2_** = *v*_2x_, *v*_2y_, *v*_2z_, applying the rotation encoded in a unit quaternion ***q***, by

(6) $$v_{2}=\;q^{*}\;⊗\;v_{1}\;⊗q=qROT(q,v_{i})$$

(For this, ***v*_1_** needs to be “cast as pure quaternion”, and ***v*_2_** must be “re-interpreted” as a vector). Additionally, computing ***v*_2_** = *qROT* (***q*_1_**, ***v*_1_**) and then ***v*_3_** = *qROT* (***q*_2_**, ***v*_2_**) will perform the rotations encoded by ***q***_1_ and then ***q***_2_, in sequence, to yield ***v*_3_** as the final result (i.e., “compounding”, where an equivalent quaternion for the compound rotation is $q_{COMPOUND}=q_{2}\;⨂q_{1}$).

Using these quaternion manipulations, GMVD will

(a) Create one orientation estimate derived from gyroscope measurements, ***q***_G_, and two estimates that are independently corrected with information from the accelerometer, ***q***_GA_, and the magnetometer, ***q***_GM_.
(b) Scale down the strength of the accelerometer- and magnetometer-based corrections by interpolating from ***q***_G_ to ***q***_GA_ and from ***q***_G_ to ***q***_GM_ using “spherical linear interpolation” (SLERP), operations [40] controlled by the corresponding trustworthiness parameters α and μ. This defines the “scaled” corrected quaternions ***q***_SA_ and ***q***_SM_, respectively.
(c) Finally, fuse ***q***_SA_ and ***q***_SM_, via a “second tier” of SLERP interpolation, to define a final MARG orientation estimation quaternion, ***q***_OUT_, which uses information from the 3 sources available, but would not contain strong corrections directed by the accelerometer or the magnetometer if their preconditions are not met.

**Figure 1:**
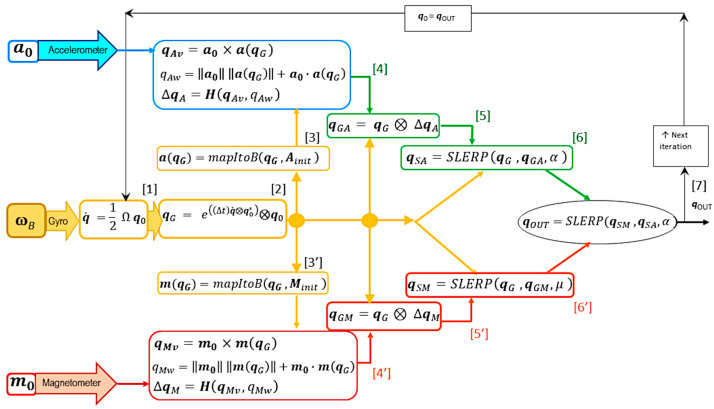
Flow of information within a single iteration of the GMVD orientation estimation algorithm. The numbers in square brackets indicate the computation steps.

The flow of information through one iteration of the GMVD algorithm is diagrammed in Figure 1. The numbers in square brackets indicate the computation of intermediate variables and the overall result, ***q***_OUT_.

At “startup”, when the orientation of the body frame in the MARG is assumed coincident with the orientation of the inertial frame, several samples of the accelerometer and magnetometer readings are collected and averaged. These initial accelerometer and magnetometer vectors, **A**_init_ and **M**_init_, must be obtained with the MARG static and in an area where the geomagnetic field is not distorted. The quaternion ***q***_NOROT_ = [0, 0, 0, 1]^T^ is assigned as the seed value of the MARG rotation estimate, ***q***_0_ = ***q***_NOROT_.

[1] The readings from the gyroscope are “de-biased” by subtracting from each of them the most recent estimate of its bias value, computed every time the MARG is estimated to be static, by linear regression on a window of consecutive samples. This yields the vector of “de-biased” gyroscope readings, **ω_B_** = [ω_Bx_, ω_By_, ω_Bz_]. (Unfortunately, under many circumstances, this “de-biasing” is not 100% successful.) Equation (7) uses the de-biased gyroscope readings to compute the quaternion rate of change:

(7) $$\dot{q}=\frac{1}{2}\;\left[\begin{array}{cccc} 0 & \omega_{Bz} & {-\omega }_{By} & \omega_{Bx}\\ {-\omega }_{Bz} & 0 & \omega_{Bx} & \omega_{By}\\ \omega_{By} & {-\omega }_{Bx} & 0 & \omega_{Bz}\\ {-\omega }_{Bx} & {-\omega }_{By} & -\omega_{Bz} & 0 \end{array}\right]\left[\begin{array}{c} q_{0x}\\ q_{0y}\\ q_{0z}\\ q_{0w} \end{array}\right]=\frac{1}{2}\;\Omega \;q_{o}$$

[2] The integration of the quaternion rate of change is completed with this quaternion expression to yield the current initial estimate of MARG orientation, which already has incorporated the latest (“de-biased”) gyroscope readings:

(8) $$q_{G}=e^{\left(\left(∆t\right)\dot{q}⨂q_{0}^{*}\right)}⨂q_{0}$$

At this point, two pipelines, one for the accelerometer correction and one for the magnetometer correction, are developed “in parallel”, as illustrated in Figure 1. As they are functionally equivalent, we outline the steps ([3]–[6]) in the accelerometer (upper) pipeline, with the comments being symmetrically applicable to the magnetometer (lower) pipeline ([3′]–[6′]).

[3] A “computed body acceleration vector”, ***a***(***q***_G_), is calculated as the mapping of the acceleration of gravity represented in the inertial frame, **A**_init_, to the current orientation of the body frame, using for this mapping ***q***_G_, obtained provisionally from just the integration of gyroscope readings:

(9) $$–a(q_{G})=\;mapItoB\left(q_{G},\;A_{init}\;\right)=q_{G}^{*}\;⊗\;A_{init}\;⊗\;q_{G}\;\;\;$$

The computed body acceleration vector should, ideally, match the current readings of the accelerometer, ***a***_0_. However, if ***q***_G_ has already developed drift, the “calculated acceleration vector” will not match the current readings of the accelerometer, ***a***_0_.

[4] It is possible [41,42] to determine the quaternion **Δ*q***_A_, representing the “rotation difference” that must be compounded with the initial ***q***_G_ into a “fully (accelerometer) corrected” estimate, ***q***_GA_, which would yield a vector matching ***a***_0_, if used to map **A**_init_ to the body frame:

(10) $$∆q_{A}=H\left(q_{Av},q_{Aw}\right)$$

where the “vector” and “scalar” parts being assembled into the quaternion **Δq**_A_ are

(11) $$q_{Av}=a_{0}\times a\left(q_{G}\right)$$

(12) $$\mathrm{and}\;q_{Aw}=\left\|a_{0}\right\|\left\|a\left(q_{G}\right)\right\|+a_{0}\cdot a\left(q_{G}\right)$$

[5] The compounding is done by this quaternion product:

(13) $$q_{GA}=q_{G}⊗∆q_{A}$$

[6] A key feature of GMVD is that it does not unconditionally accept this “fully” accelerometer-corrected quaternion as a better estimation of the MARG orientation. While ***q***_GA_ is, indeed, infused with information from the instantaneous accelerometer readings, which may correct gyroscopic drift that could have accumulated, it may, itself, be subject to error. That would be the case if the current accelerometer measurements, ***a***_0_, are not just measuring the acceleration of gravity, which is the precondition assumed to interpret ***a***_0_ as a body frame observation of the same vector recorded in **A**_init_ referenced to the initial orientation, i.e., to the inertial frame. Thus, a “scaled” accelerometer-corrected orientation, ***q***_SA_, is found interpolating from ***q***_G_ to ***q***_GA_ under control of the accelerometer trustworthiness parameter 0 ≤ α ≤ 1, using SLERP interpolation. If the accelerometer preconditions are completely absent (i.e., the MARG is moving at variable speed), α will be close to 0, and the interpolated ***q***_SA_ will essentially be ***q***_G_ (no harm done). If the accelerometer preconditions are met (α ≈ 1), then ***q***_SA_ will be, essentially, ***q***_GA_. That is, the system will take full advantage of the valid secondary source of MARG orientation information.

(14) $$q_{SA}=SLERP(q_{G}\;,\;q_{GA},\;\alpha )$$

In general, the SLERP interpolation from quaternion ***q***_1_ to quaternion ***q***_2_, under control of the parameter 0 ≤ *h* ≤ 1, is computed as [40]

(15) $$cos\left(\Omega \right)=q_{1}\cdot q_{2}$$

(16) $$SLERP(q_{1},q_{2},h)=\frac{q_{1}sin((1-h)\Omega )+q_{2}sin(h\Omega )}{sin(\Omega )}$$

In the magnetometer pipeline, step [6′] implies the SLERP interpolation from ***q***_G_ to a “fully” magnetometer-corrected estimate ***q***_GM_, under control of the magnetometer trustworthiness parameter 0 ≤ μ ≤ 1. This step has the same objective as step [6]. In this case, μ quantifies the level of fulfillment of the magnetometer precondition. The current magnetometer measurements, ***m***_0_, qualify as an observation of the same vector as **M**_init_ only if the geomagnetic field at the current location of the MARG is undistorted. The computation of the trustworthiness parameters α and μ is explained in the following subsection.

[7] Finally, the 2 corrected MARG orientation estimates which potentially took advantage of accelerometer and magnetometer readings, but have curtailed strong corrections if their preconditions were not actually met, are fused with a second-tier SLERP interpolation:

(17) $$q_{OUT}=SLERP(q_{SM},q_{SA},\alpha )$$

### 2.2. Computation of the Trustworthiness Parameters α and μ

The overall scheme of the GMVD algorithm requires that values of the accelerometer and magnetometer trustworthiness parameters, α and μ, be available for every sampling interval of operation of the MARG. The α parameter must estimate how close to static (or moving without linear acceleration) the MARG might be, so that the current accelerometer readings can be interpreted as a good approximation of gravity components along the body frame axes. The μ parameter must represent how close the magnetic field currently measured by the magnetometer is to the undistorted geomagnetic field first measured at startup.

#### 2.2.1. Original Formulations

Our computation of α was based on a parameter internally calculated by the MARG module we have used (Yost Labs 3-Space Micro USB), called “confidence (factor)”, directly readable (along with the accelerometer, gyroscope, and magnetometer values) at every sampling interval [43]. However, we have confirmed that a similar parameter could also be calculated based on the rotational and translational activity of the MARG as a normalized average of the mean squared values of the gyroscope and accelerometer readings. In either case, the original parameter value is processed by a linear equation whose negative slope was set to produce a rapid decrease in the value of α as soon as the MARG begins to depart from a static status. In our intended use of MARG modules for tracking hand-held human–computer interaction devices, it is reasonable to expect that there will be frequent static intervals, in which α will increase to values close to 1. (After the linear function, we also overwrite all negative results to zero, which is the lower bound of the desired values of α, with this conversion: α = (α + |α|)/2.)

The computation of the μ parameter is more involved, as it should represent a property of the environment in which the MARG is currently operating, and not one related to its own motion. Under the assumption that the large ferromagnetic objects around the human body being monitored will not be in constant and rapid motion, we previously sought to model the distortion of the geomagnetic field as a property of three-dimensional space. In a previous implementation of a system for tracking the position and configuration of the hand of a human subject [44,45], the MARG was used in conjunction with a 3-IR camera system (OptiTrack V-120 Trio. OptiTrack is a division of NaturalPoint, Inc., Corvallis, OR, USA), that provided X, Y, and Z position coordinates for the MARG. This previous version of the algorithm, designated as the “Gravity and North Vector Correction with Double Slerp” (GMVD) orientation estimator, used the position information provided by the camera system to manage a 3D map of the space where the MARG is moving, populating small (e.g., 2 cm per side) cubic “voxels”, with estimated values of the μ parameter. The computation of μ in the GMVD algorithm details have been documented in [42] and will not be recounted here, as we have now superseded it with the self-contained μ computation detailed next.

#### 2.2.2. New Computation of Magnetometer Trustworthiness, μ_K_, without Position Information (GMVDK)

Aware of the existence of application scenarios where the use of a camera system to aid in the storage and retrieval of previously computed μ values may not be practical, we have now developed an alternative magnetometer trustworthiness parameter, μ_K_, which is *calculated without involvement of MARG position information*. We seek to encode in 0 ≤ μ_K_ ≤ 1 the trust we can have that the geomagnetic field at the current location of the MARG is the same as the one initially sensed at startup, **M**_init_. If the magnetic vector is the same in magnitude and direction (μ_K_ ≈ 1), then the precondition for the magnetometer-based correction to the orientation estimate is met and it can be applied fully. If the precondition is not fully met, the magnetometer-based correction must be restrained accordingly.

As mentioned previously, the system stores the components of the initial magnetic vector, **M**_init_, referenced to the initial orientation of the MARG body frame, which coincided with the inertial body frame at startup. The magnetic vector currently sensed by the MARG, ***m***_0_, may differ from the initial, **M**_init_, with respect to magnitude and direction. Our approach first computes two parameters that focus primarily on each of these possible alterations: μ_KA_ (based on angle change) and μ_KM_ (which also uses magnitude change). However, ***m***_0_ is referenced to the body frame, whereas **M**_init_ is referenced to the inertial frame. To compare them properly, we can “map” the current readings of the magnetometer, ***m***_0_, “back” to the inertial frame. Here, we take advantage of the limited rotational speed expected in the movement of a body segment to approximate the current orientation of the MARG body frame with the final orientation estimate obtained in the previous iteration: ***q***_OUT_. Thus, the magnetometer readings mapped back to the inertial frame will be

(18) $$m_{0i}=\;mapBtoI\left(q_{OUT},\;m_{0}\;\right)=q_{OUT}\;⊗\;m_{0}\;⊗\;q_{OUT}^{*}\;\;\;$$

The angle, λ, between the 2 vectors is

(19) $$\lambda =\cos^{-1}\left[\frac{M_{init}\cdot m_{0i}}{\left\|M_{init}\right\|\left\|m_{0i}\right\|}\right]$$

The larger the value of λ, the more distorted the magnetic field is at the current position of the MARG. So, μ_KA_ is formulated to drop quickly from the value of 1 that it adopts when λ is 0 (negative values of μ_KA_ are overwritten with 0):

(20) $$\mu_{KA}=1-1.5\;\lambda$$

Changes in magnitude help detect distortion of the magnetic field at the current MARG location but will only be detrimental if there is a simultaneous change in orientation of the magnetic field vector. Thus, μ_KM_ was set to decay from its ideal value of 1 by a “penalty” value given by

(21) $$penalty=\left[\frac{\left\|m_{0i}\right\|}{\left\|M_{init}\right\|}\right]\;\left(\lambda \right)$$

(22) $$\mu_{KM}=1-penalty$$

and negative results for μ_KM_ are overwritten with a value of 0. The 2 parameters previously described are consolidated as

(23) $$\mu_{1}=\left[\mu_{KA}+\mu_{KM}\right]/2$$

To enforce a continuous suppression of magnetometer corrections when μ_1_ is varying rapidly, μ_2_ is obtained as the minimum value of μ_1_ in the present and past values computed in the last 0.4 s. The final trustworthiness parameter is

(24) $$\mu_{K}=\left(\mu_{2}\right)\left(\alpha \right)$$

to discourage magnetometer corrections when the MARG might be in rapid rotation (small α), as that would violate our approximation of the current MARG orientation by the immediately past ***q***_OUT_.

#### 2.2.3. Pseudocode for Parameter Computation and Parameter Sensitivities

The main flow of information depicted in Figure 1 can be implemented unambiguously by programming Equations (7)–(17). On the other hand, the computation of the trustworthiness parameters α and μ_K_, can be characterized more clearly through the corresponding pseudocode programs presented in Appendix B. Further, each of the pseudocode programs (Appendix B.1 and Appendix B.2) include a parameter. For the computation of α, the parameter is “SlopeAlpha”, and for the computation of μ_K_, the parameter is “SlopeMuk”. In each case, the larger the value of the parameter, the more sensitive the corresponding parameter (α or μ_K_) will be to departures from the corresponding ideal condition (complete lack of movement for α and complete lack of magnetic field distortion for μ_K_). After examination of the behavior of GMVDK in multiple cases, with several values of these parameters, our descriptions in Section 2.2.1 and Section 2.2.2 above use the values of these parameters we selected as “nominal values”: SlopeAlpha = 1.0 and SlopeMuk = 1.5. Nonetheless, it was of interest to study to what degree the performance of GMVDK might be changed if these parameters are assigned other values—in other words, the sensitivity of GMVDK performance to changes in these parameters. The description of our sensitivity assessment process and the results are detailed in Appendix B. In summary, we found that for a 1% change in SlopeAlpha, the change in GMVDK performance, as defined in our experiment, is of the order of 0.0856%. We also found that for a 1% change in SlopeMuk, the change in GMVDK performance is of the order of 0.1002%. These results seem to indicate that the precise use of the nominal parameter values is not critical to obtain similar levels of performance from GMVDK.

## 3. Results

In this section, we include detailed descriptions of the results obtained from two representative runs of an experimental sequence of manipulations of the 3-Space Micro USB MARG module from Yost Labs (the complete set of specifications offered by the manufacturer for this MARG module are transcribed in Appendix A). In both examples, we show the orientation estimates generated by our new GMVDK algorithm (i.e., GMVD with μ_K_), which does not require position information from the system of infrared cameras. In the first example, we compare the results from GMVD (previous version) and GMVDK to orientation estimates generated internally by the MARG according to a Kalman filter algorithm implemented in the chip by the manufacturer. In the second example, we contrast our results with those from two well-known contemporary MEMS MARG orientation estimation algorithms.

### 3.1. Experimental Protocol

**Figure 2:**
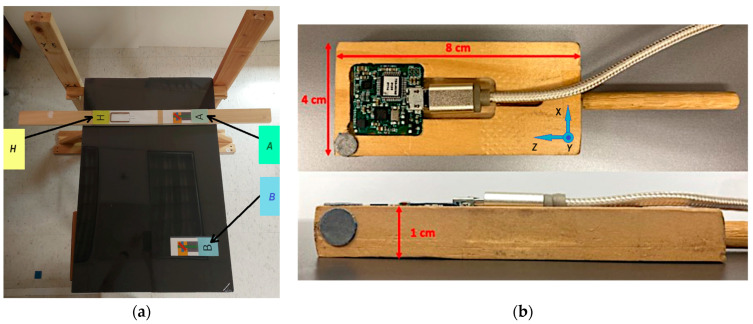
Recording setup used to perform the rotation sequence described in Table 2. (**a**) MARG recording space (top view); (**b**) 3-Space Micro USB MARG mounted on a wooden prism.

We present the orientation estimates obtained by GMVDK from the Yost Labs 3-Space Micro USB MARG module [43] , affixed to a small wooden prism, as it was manipulated by a human subject. This was adopted so that the MARG could be placed in repeatable orthogonal orientations by resting the prism on a level surface. During the trial, the MARG was manipulated at three locations. Locations H, A, and B were set at an approximate height of 1 m from the floor. A and B were located on a line approximately oriented west–east. B was located approximately 55 cm west from A. The home location (H) was approximately 30 cm north from A. Whereas all the supporting surfaces used for the experiment were made of wood and placed away from steel or iron furniture, a 0.5 × 3.8 × 37.5 cm steel bar was placed directly under Location B, to have a known distortion of the magnetic field around that location. Figure 2 shows a top view of the plane in which locations H, A, and B were defined and the wooden prism on which the 3-Space Micro USB MARG was mounted.

A human subject completed a pre-specified sequence of rotations involving the same orientation changes performed first at Location A, where the geomagnetic field was not distorted, and then at Location B, where the magnetic field was known to be distorted. In between rotations, the subject was asked to hold specific “poses”. The pre-defined sequence is indicated in Table 2 (rotations are designated according to the “left-hand rule”).

The main means of verification for the resilience of GMVDK to magnetic distortion is the similarity of the orientation estimation results obtained for the same sequence of poses (Poses 1–5) adopted first in an environment where the geomagnetic field is not distorted (Location A) and then (Poses 6–10) in a distorted magnetic field (Location B).

### 3.2. Orientation Estimates Obtained from Different Approaches

**Figure 3:**
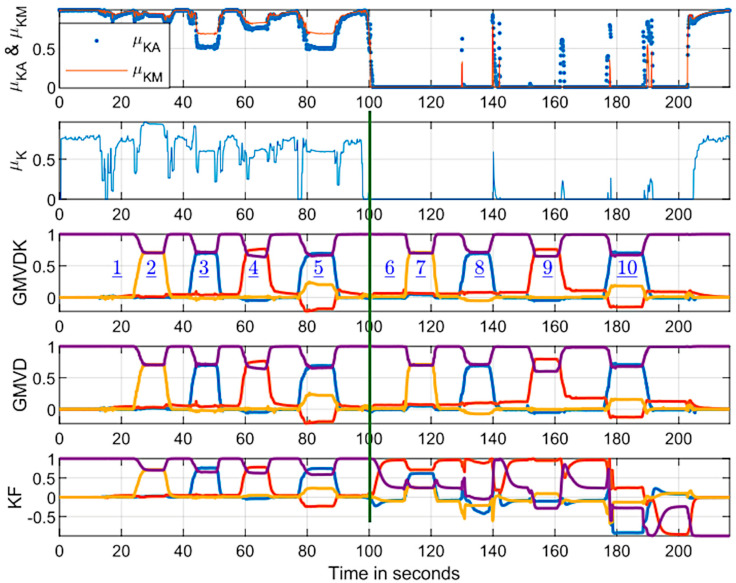
New μ_K_ trustworthiness parameter and results from three orientation estimation algorithms. All the panels show the evolution of the corresponding values through time. From top to bottom: (1) the two μ_K_ components: μ_KA_ and μ_KM_; (2) the alternative magnetic trustworthiness parameter, μ_K_; (3) the 4 components of the quaternion generated by GMVDK (using μ_K_); (4) the 4 components of the quaternion from the previous algorithm version (GMVD); (5) the 4 components generated by the internal Kaman filter (KF) in the MARG module used. The blue underlined numbers are the corresponding pose numbers. The components of all quaternions are color-coded as follows: x in blue; y in red, z in yellow and w in purple.

Figure 3 shows the evolution of the orientation estimates obtained by GMVD (previous version requiring X, Y, and Z position information from the 3-IR camera system, which was recorded for this first experiment) and by our new algorithm, GMVDK. Figure 3 also shows the orientation estimates from the Kalman filter (“KF”) implemented within the Yost Labs MARG. This result is included to facilitate the comparison of our method to an orientation estimation algorithm that is well known and has been used extensively for orientation estimation [46,47,48,49]. In each case, the orientation estimation is represented by the four quaternion components, which are shown as they evolve through time. For clarification, a vertical green line has been included at the approximate time when the MARG was translated from Location A (not magnetically distorted) to Location B (magnetically distorted). For reference, Figure 3 also includes the two components of the new µ_K_ (i.e., µ_KA_ and µ_KM_), overlapped, and a plot of the evolution of µ_K_ itself, which confirms its significant decrease when the MARG was translated from Location A to Location B and its increase again when the MARG was finally translated back to the home location (H).

The plots in Figure 3 show how, while all three approaches, GMVD, GMVDK, and KF, report very similar orientation estimates for all the poses held in Location A (not magnetically distorted), the results generated by KF are markedly different for the same poses at Location B (magnetically distorted).

On the other hand, both GMVD and GMVDK report orientation results that are very similar in Location A and in Location B, confirming their resilience to the distortion of the geomagnetic field.

**Figure 4:**
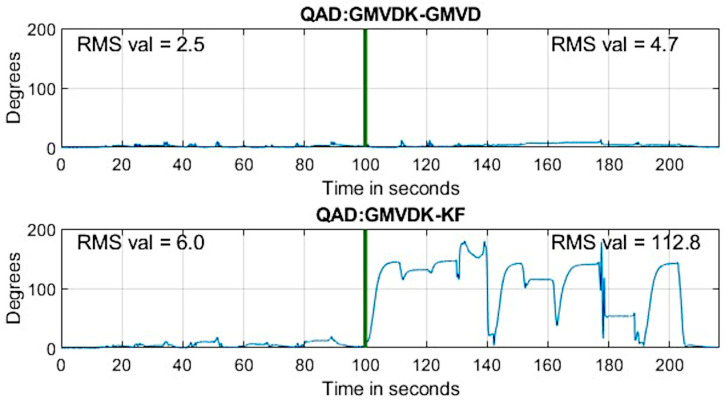
Quaternion angle difference (QAD) between GMVDK and GMVD (**top** panel) and quaternion angle difference (QAD) between GMVDK and KF (**bottom** panel). In each case, the RMS value of QAD for the intervals before and after the vertical dividing line is indicated.

To assess the orientation differences between results from our proposed GMVDK and the traditional Kalman filter estimator, the quaternion angle difference (QAD) between these quaternions was computed at every sampling instant, using the “dist” command from Matlab^®^ [50], which we verified yields the same results as the formulation to obtain the QAD between quaternions ***q***_a_ and ***q***_b_ presented in [34]:

(25) $$QAD\left(\;q_{a},\;q_{b}\right)={cos}^{-1}\left(2\;\langle q_{a}\;\cdot \;q_{b}\rangle -1\right)$$

where <***q***_a_ ∙ ***q***_b_> represents the quaternion inner product. We also computed the QAD between orientations from GMVDK and our previous formulation, GMVD. Figure 4 shows the evolution of both those quaternion angle differences. The top panel shows that both our formulations were fairly consistent, displaying minimal QAD throughout. In this top panel, the change in QAD from the first segment of the recording, acquired at Location A, and the second segment of the recording (to the right of the vertical dividing line), acquired at Location B, was very small. The computed RMS values for the segments before and after the dividing line are included in the panel. This reveals that both GMVD and GMVDK performed similarly in the magnetically distorted environment (Location B) and in an environment that was not affected by magnetic distortion (Location A). Since the orientation sequences performed at Location A and at Location B were the same, the top panel of Figure 4 and the traces for GMVD and GMVDK in Figure 3 lead us to the conclusion that both GMVD and GMVDK displayed robustness to the magnetic distortion that existed in Location B.

On the other hand, the onboard Kalman filter algorithm provided results that are also very close to the ones from GMVDK when the MARG was at Location A (recording a low QAD RMS of 6.0°) but yielded markedly different orientation results when the MARG was at Location B, resulting in large QAD differences with GMVDK in some instants and, overall, reporting a very high QAD RMS of 112.8° after the vertical line (i.e., while the MARG was at Location B).

**Figure 5:**
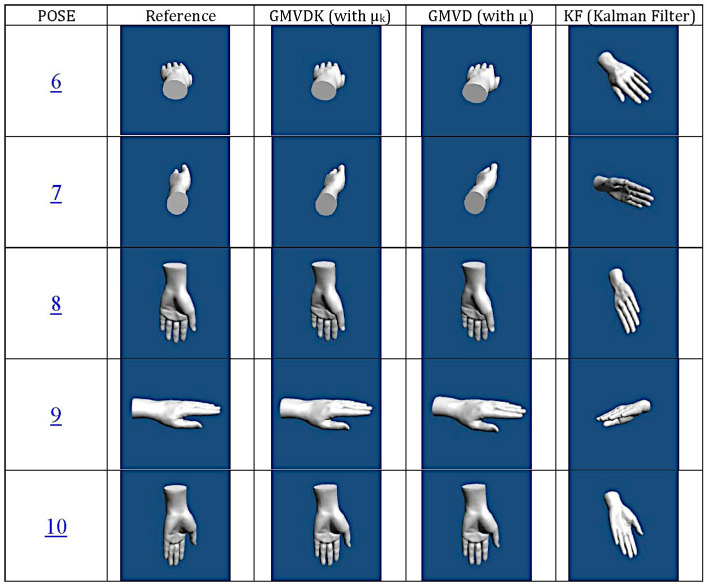
Three-dimensional visualization of the orientations obtained from 3 MARG orientation estimation algorithms at the times indicated, with the corresponding blue underlined numbers in the GMVDK panel of Figure 3. From left to right: (1) pose number; (2) visualization of the reference orientations; (3) visualization of the orientations generated by GMVDK; (4) visualization of the orientations generated by GMVD; (5) visualization of the orientations generated by the Kalman filter (KF).

In Figure 3, the marked change of the four KF traces before and after the dividing line makes it evident that this estimator was impacted by the magnetic distortion at Location B. But the impact of the errors generated was much better appreciated by 3D visualization of an object oriented according to the estimates. Figure 5 shows renderings of a hand model created in Unity^®^ that has been oriented using the quaternion values yielded by GMVD, GMVDK, and by KF at poses 6, 7, 8, 9, and 10, as identified by the blue underlined numbers inside the GMVDK plot in Figure 3. The leftmost column of renderings shows the hand oriented according with ideal or “reference” orientations that were instructed to the subject (i.e., as the orientations would have been without any error). The error in the orientations estimated by KF ranged in severity, but they did include instances where the reference hand and the KF-oriented hand showed markedly different orientations.

We also had an interest to compare the results of processing the same MARG signals by GMVDK and by two other contemporary MARG orientation estimation approaches. Therefore, we up-sampled data collected in a second representative run (originally collected at 15 samples per second) to match the expected 256 samples per second in the Matlab^®^ (Version R2023a) implementations of Madgwick’s [51] and Mahony’s [52] MARG orientation estimation algorithms posted by S. Madgwick (at https://x-io.co.uk/downloads/madgwick_algorithm_matlab.zip, accessed on 16 January 2024). We set the adjustable parameters involved in each of the two algorithms to the same values used in the examples provided with the implementation code (Beta was 0.1 for Madgwick’s and Kp was 0.5 for Mahony’s).

**Figure 6:**
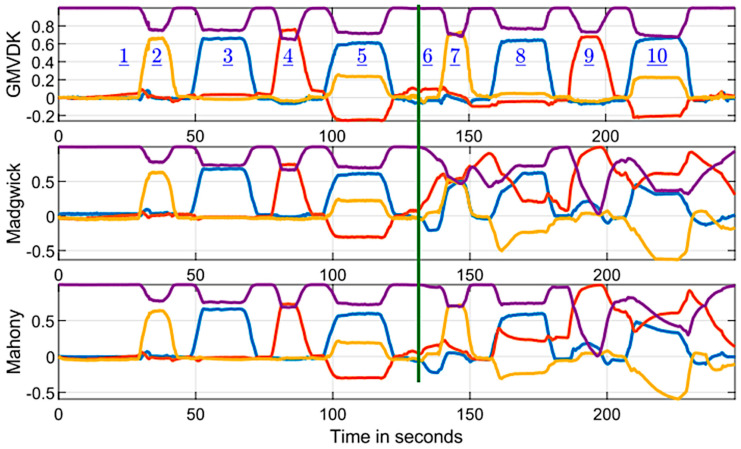
Comparison of results from GMVDK and 2 alternative approaches. All three panels display the 4 components of the corresponding orientation quaternion as they evolve through time. From top to bottom: (1) GMVDK; (2) Madgwick’s algorithm; (3) Mahony’s algorithm. The blue underlined numbers are the corresponding pose numbers. The components of all quaternions are color-coded as follows: x in blue; y in red, z in yellow and w in purple.

Figure 6 shows the resulting comparison between the algorithm results for this second experimental trial (also following the pre-specified sequence of actions described in Table 2). As it happened for KF, the results from all the algorithms while at Location A (left of the green vertical dividing line) were very similar. However, once the MARG was translated to Location B (right of the green vertical dividing line), GMVDK generated outputs that were very close to the ones obtained for the same sequence in Location A, but both Madgwick’s algorithm and Mahony’s algorithm generated very different quaternions.

**Figure 7:**
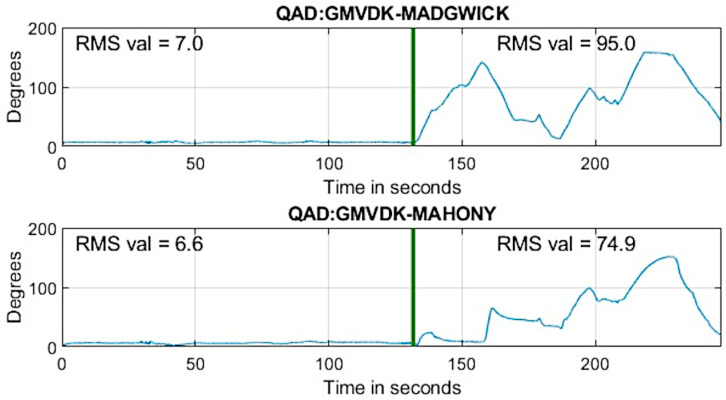
Quaternion angle difference (QAD) between GMVDK and Madgwick’s algorithm (top panel), and quaternion angle difference (QAD) between GMVDK and Mahoney’s algorithm (bottom panel). In each case, the RMS value of QAD for the intervals before and after the vertical dividing line is indicated.

Following the same analysis as we used to create Figure 4, Figure 7 shows the evaluation of the quaternion angle difference between GMVDK and the Madgwick algorithm (top panel) and between GMVDK and the Mahony algorithm (bottom panel). In both cases, the observations were similar to the ones from Figure 4. Both algorithms yielded orientation estimates that were close to those from GMVDK while the MARG was at Location A (to the left of the vertical dividing lines in Figure 7), recording low RMS QAD values of 7.0° and 6.6°. However, when the MARG was manipulated in the magnetically distorted environment at Location B (to the right of the vertical dividing lines in Figure 7), the orientations reported by the Madgwick and Mahony algorithms were significantly different to the orientations reported by GMVDK, recording high RMS QAD vales of 95.0° and 74.9°, respectively.

Since the sequence of rotations executed at Location A and at Location B were the same, the markedly different sequences of quaternion components in the second half of the recording generated by the Madgwick and Mahony algorithms led to the conclusion that these two algorithms were much more affected by the magnetic distortion than GMVDK. Further, the QAD differences between GMVDK and each of the other two methods in the right half of Figure 7 provide an approximate quantification of those degradations. Overall, these comparisons support our belief that GMVDK has higher resilience to distortions in the magnetic field.

### 3.3. Additional Evaluations of GMVDK Performance

Beyond the comparisons to alternative orientation estimation methods described in the previous subsection, it was of interest to assess other specific aspects of the performance of the GMVDK algorithm.

Two important aspects to verify in any MARG orientation estimation algorithm are their suitability for real-time implementation in an ordinary computation platform, at a reasonable sampling rate, and their longer-term stability. To determine the execution time required by one iteration of the GMVDK algorithm, on data obtained from one MARG module, we used a real-time C-sharp implementation of GMVDK in which we programmed the recording of a timestamp to a file both just before and immediately after the group of instructions that implement GMVDK. Computing the time difference between 180,000 of those pairs of timestamps, we computed the execution time mean value to be 0.38 milliseconds, with a standard deviation of 0.24 milliseconds. This testing was performed in an ASUS TUF Dash F15 (2021) personal computer, with an 11th Gen Intel Core i7-11370H processor, at a clock rate of 3.30GHz. The mean GMVDK execution time of 0.38 milliseconds indicates that this commodity platform could compute up to 2628 GMVDK iterations per second. Sampling rates commonly used for MARG sensors in the context of human–computer interactions (e.g., 100 Hz) would establish sampling intervals (e.g., 10 milliseconds) that are much longer that the 0.38 millisecond execution time recorded for GMVDK. Accordingly, the execution time of GMVDK seems appropriate for real-time implementation.

To investigate the level of long-term stability that can be expected from the GMVDK algorithm, we performed an overnight, 10 h recording of the quaternion results using the same real-time C-sharp program, running on the same personal computer as described in the previous paragraph. The program logged to file the quaternions from both the onboard Kalman filter and from GMVDK. During this recording, the MARG module was kept static, such that the quaternion expected as the result from both algorithms was quaternion ***q***_NOROT_ = [0, 0, 0, 1]^T^. The quaternion values obtained from both algorithms were, indeed, very close to the expected quaternion ***q***_NOROT_. We evaluated the minor deviations from the expected value by computing the QAD between the ***q***_NOROT_ and the GMVDK quaternion for all sampling instants (over the 10 h recording length) and then obtaining the root-mean-squared value of the QAD sequence. We obtained a value of RMSE = 0.4447° for GMVDK. Computing the same metric for the onboard Kalman filter, we obtained RMSE = 2.5291°. This confirmed that GMVDK was, on average, able to keep the orientation errors for this long-term static test to less than half a degree, with the onboard Kalman filter displaying an RMSE value that was bigger.

We also sought to find out if GMVDK would exhibit resilience to different intensities and directions in the magnetic disruption. To study this, the magnetic disruptor was placed 15 cm to the south of Location B (where the second repetition of manipulations resulting in Poses 6 through 10 were still executed), and we verified that GMVDK was less impacted by the magnetic distortion than the Kalman filter under these conditions as well. The details related to this experiment are included in Appendix C, where a second experiment in which the magnetic disruptor was placed 15 cm north from Location B is also described.

Lastly, we studied the ability of GMVDK to provide appropriate estimates of the orientation of the MARG after it had been subjected to a magnetically distorted environment. We did this by translating the MARG from the home location (H) to the magnetically disturbed Location B first and holding the five poses there. After the five poses were completed in Location B, the MARG was translated to the magnetically undisrupted Location A, where the five poses were repeated and then the MARG was translated back to the home location (H). In this so-called H-B-A-H route experiment, we found that GMVDK was able to yield correct orientation estimates for the poses held at Location A immediately, whereas the Kalman filter continued to yield inconsistent orientation estimates for a few milliseconds after the MARG was removed from Location B. A detailed account of this experiment and its results is presented in Appendix D.

## 4. Discussion

Early in this century, the capabilities of the MEMS accelerometer and gyroscopes in MEMS IMUs were found to be insufficient for use in the strapdown configuration for simultaneous position and orientation tracking (e.g., [8]). This led to a narrowing of the goals pursued by using these devices (traying to track orientation only) and to the recruitment of a third kind of MEMS sensor (tri-axial magnetometer) in the effort to provide useful estimates of the orientation of the resulting MARG modules.

Undoubtedly, having the availability of magnetometer signals represents an additional infusion of information to the estimation process, and multiple algorithms have been designed with the implicit aim to fully utilize the information from all three different types of signals (angular rate of change, acceleration, and magnetic field measurements). It may be, however, that too much of an emphasis has been placed in the inclusion of available signals, without enough vigilance regarding the instances in which the information from some of the signals, while available, should better be excluded, or at least restrained.

This began to be realized by the users of MARG orientation estimation algorithms, as noted in the 2009 paper by DeVries et al. [26], where they alerted the biomechanics research community about the possibility of degradation of orientation estimates obtained from some MEMS modules due to geomagnetic disturbances. In that article, they suggested that researchers using this type of MEMS modules for studies tracking the motion of limbs in human subjects should “map” the magnetically distorted areas of their laboratories and avoid using them if MEMS MARG modules were involved. This confirms the severity of the consequences that ignoring magnetic disturbances could have in biomechanical studies.

We have proposed the GMVDK algorithm, described in Section 2, with an emphasis on restraining the involvement of corrections to the initial gyroscopic dead reckoning MARG orientation estimation based on magnetometer or accelerometer signals, if our algorithm finds that the corresponding preconditions are not fully met. While our approach still has the prediction–correction sequence that also exists in Kalman filters, we apply the corrections based on accelerometer and magnetometer information separately, and only to the extent that the corresponding trustworthiness parameter indicates that the correction should be applied. The results presented in Section 3 seem to support our view that GMVDK is more resilient to the degradation of the orientation estimates caused by magnetic disturbances than three other approaches: Kalman filter, Madgwick’s algorithm, and Mahony’s algorithm.

We designed our experimental protocol so that the same sequence of rotations and poses were performed in both a magnetically undistorted environment (Location A) and a magnetically distorted environment (Location B). Therefore, any significant difference in the output from an algorithm while at Location A in contrast to its output at Location B indicates that the algorithm was impacted by location. By construction, the only important difference between the locations in our setup was the absence/presence of magnetic distortion in Location A/Location B. Therefore, we interpret our observations (Figure 3 and Figure 6) to mean that GMVDK was less affected by magnetic distortion than the Kalman filter, Madgwick’s algorithm, and Mahony’s algorithm, which is more plainly visualized by the QAD plots contained in Figure 4 and Figure 7. Further, 3D renderings of a virtual object, such as the hand shown in Figure 5, make the degradation of the Kalman filter estimate easier to perceive and gauge intuitively.

The results from both experiments in Figure 4 and Figure 7 seem clear with respect to the strong impact of the conditions at Location B on the orientation estimates produced by the alternative estimation algorithms. In the absence of externally determined ground truth for the orientation in these experiments, we have used the GMVDK estimate as an approximation to the expected correct estimates of orientation. This is because the behavior of the GMVDK estimates at Location A and at Location B, in both Figure 3 and Figure 6, is very similar, as would be expected ideally. Accordingly, we present the QAD comparing the estimates from GMVDK to those from the Kalman filter (Figure 4) and to the estimates from the Madgwick and Mahony algorithms (Figure 5) to quantify the degradation of these alternative methods. We observe very significant changes in the RMS values of those quaternion angular differences before and after the MARG was transitioned from Location A to Location B: Kalman filter: 6° to 112.8°; Madgwick method: 7° to 95°; and Mahony Method: 6.6° to 74.9°.

One additional important observation is that the recurrent nature of Madgwick’s and Mahony’s algorithms caused them to still yield erroneous orientation estimates even in the last interval of the recording (Figure 6), even when the MARG had already been returned to the home (H) position, where the magnetic field was undistorted. Figure 7 shows that even when the MARG had been translated to a location without magnetic distortion (H), these algorithms were only slowly (progressively) reducing their error, as indicated by the corresponding QAD plots. This further highlights the importance of preventing inappropriate magnetometer-based corrections from taking place in the first place. This is because once such errors enter a recurrent estimator, they are likely to degrade the results for the current iteration and also for future iterations.

## 5. Conclusions

When low-cost miniature MEMS accelerometers and gyroscopes became commercially available, several research communities (biomechanics, human–computer interaction, etc.) wished that they could replicate the capabilities of larger, navigation-grade IMUs. In particular, there was hope that they could be used for tracking the position and orientation of articulated segments of the human body in direct or indirect (e.g., tracking hand-held devices) ways. Micromachining technology had created these miniature, low-power, low-cost devices that were able to produce measurements of accelerations and rotational speeds and the expectations were initially very high.

Unfortunately, even today, the use of these MEMS sensor modules (which now, with the addition of MEMS magnetometers, are consolidated into MARG modules) to confidently track orientation (alone) is challenged by the need to periodically correct orientation estimates initially generated by the gyroscope information. Multiple approaches for efficiently implementing those accelerometer- and magnetometer-based corrections have been proposed through the last few decades, but a definitive answer to this problem remains elusive. In recent years, with the emergence of the IoT, it has become clear that this is also a new and expanding area of application which could benefit from the use of MEMS MARG modules for tracking the orientation of many IoT devices.

In this paper, we presented the “Gravity and Magnetic North Vector, with Double SLERP and μ_K_“ (GMVDK) algorithm as our proposed approach to the problem, within the operational context of a human–computer interaction application (which may be the same context in which many potential IoT devices would operate).

A key difference in our approach is that GMVDK deliberately implements steps in which the prospective corrections to the initial results of gyroscope signal integration can be “restrained”. In fact, the potential accelerometer- or -magnetometer-based corrections are only applied to the extent that the algorithm “trusts” that the preconditions implicitly invoked for the design of the corrections are met.

Therefore, GMVDK also follows the prediction–correction philosophy present in the Kalman filter, but it implements it in a completely different way. In particular, an effort was made in the development of GMVDK to represent the “trustworthiness” of the prospective accelerometer- and magnetometer-based corrections in a single-scalar parameter for each. This was coupled with the consistent representation of orientations (rotations from the initial orientation) as quaternions, leveraging the use of the SLERP interpolations which can partially correct orientations under control of a single scalar parameter.

In this paper, we detailed the new way in which we compute the magnetometer trustworthiness parameter, *μ*_K_, which no longer requires information provided by any external device. Furthermore, we verified the resilience of GMVDK to magnetic distortions that can be found in modern dwellings, offices, and laboratories.

Our verification procedure showed that GMVDK produced the same (quaternion) orientation estimates for a given sequence of MARG orientations (“poses”) when it was executed in a magnetically disrupted area (Location B) as when it was executed in a magnetically undisturbed area (Location A). On the other hand, the same accelerometer, gyroscope, and magnetometer data were processed by three other well-known orientation estimators (Kalman filter, Madgwick’s and Mahony’s algorithms), yielding markedly different results in the magnetically disturbed location, which we interpreted as a degradation of the corresponding estimator performance. We visualized the difference between the results from GMVDK and the alternative estimators by plotting the quaternion angle differences at every sampling instant. Further, we quantified the differences through the RMS aggregate measurements for each QAD plot before and after the MARG was translated from the magnetically undistorted location to the magnetically distorted location.

Overall, the experimental results appear to support our belief that GMVDK provides MARG orientation estimates that are more resilient to the presence of magnetic distortions in the operating space of the MARG than the three alternative orientation estimators.

The degradation in the performance of MARG orientation algorithms due to magnetic distortions is an important challenge that must continue to be studied. We have recently created a freely accessible dataset of MARG signal files with segments that include magnetic distortion, for that purpose [53]. It is our hope that the added robustness exhibited by GMVDK might contribute to a more widespread and successful usage of MEMS MARG modules for a wider range of applications in diverse fields, including human–computer interaction.

## Figures and Tables

**Table 1:** Parameters of gyroscopes in different categories (grades) of IMUs *.

Parameter	Commercial Grade	Tactical Grade	Navigation Grade	Strategic Grade
Gyro bias initial uncertainty	**150°/h**	1.5°/h	0.0015°/h	0.0000015°/h
Gyro bias stability	$1500{}^{\circ}/h/\sqrt{h}$	$15^{o}/h/\sqrt{h}$	$0.015/h/\sqrt{h}$	$0.000015/h/\sqrt{h}$

* Values extracted from Figure 8.4 in [7]. “Commercial Grade” is underlined and bolded because it is the type that would be used in an instrumented glove for human-computer interaction.

**Table 2:** Sequence of steps in each experimental trial.

Sequence Step	Location	Rotation	Resulting Pose
1	H	(Initial location and pose for the task)	1
2	A	After translation H to A, yields	1
3	A	+90° Z axis, yields	2
4	A	−90° Z axis, yields	1
5	A	+90° X axis, yields	3
6	A	−90° X axis, yields	1
7	A	+90° Y axis, yields	4
8	A	−90° Y axis, yields	1
9	A	−45° Y axis and + 90° X axis, yields	5
10	A	−90° X axis and + 45° Y axis, yields	1
11	B	Just translation A to B	6 (same orientation as 1)
12	B	+90° Z axis, yields	7
13	B	−90° Z axis, yields	6
14	B	+90°X axis, yields	8
15	B	−90° X axis, yields	6
16	B	+90° Y axis, yields	9
17	B	−90° Y axis, yields	6
18	B	−45° Y axis and + 90° X axis, yields	10
19	B	−90° X axis and + 45° Y axis, yields	6
20	H	Just translation back to H	1

**Figure A1:**
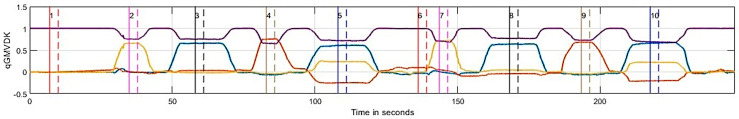
Orientation estimations from GMDVK and Kalman filter for the same case as described in Figure 6, but also indicating the time segments where reference quaternions for the poses (numbered) were known (pairs of vertical lines; solid for start and dashed for end of each interval).

**Figure A2:**
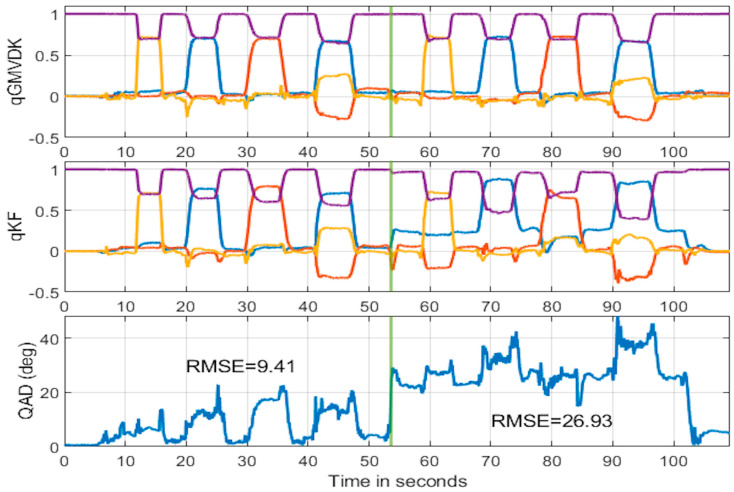
Quaternions generated by GMVDK and the onboard Kalman filter when the magnetic disruptor was placed 15 cm south of Location B, and their quaternion angle difference. The RMS values for the QAD before and after the green dividing line are included in the lower panel. The components of all quaternions are color-coded as follows: x in blue; y in red, z in yellow and w in purple.

**Figure A3:**
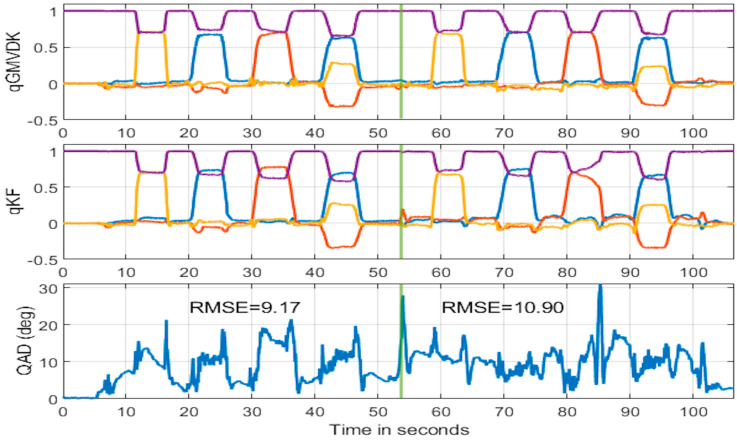
Quaternions generated by GMVDK and the onboard Kalman filter when the magnetic disruptor was placed 15 cm north of Location B, and their quaternion angle difference. The RMS values for the QAD before and after the green dividing line are included in the lower panel. The components of all quaternions are color-coded as follows: x in blue; y in red, z in yellow and w in purple.

**Figure A4:**
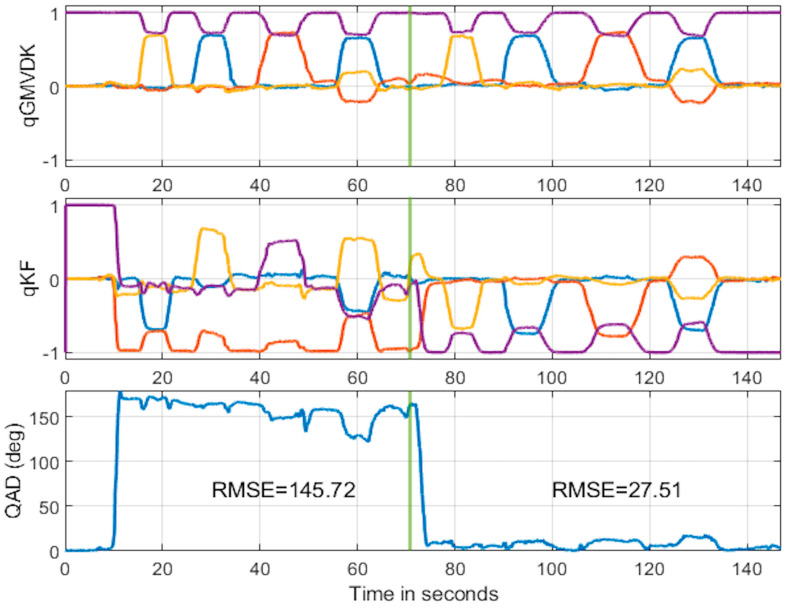
Quaternions generated by GMVDK and the onboard Kalman filter when a series of 5 poses was first executed in the magnetically disrupted Location B. The RMS value for the QAD before and after the green dividing line are included in the lower panel. The components of all quaternions are color-coded as follows: x in blue; y in red, z in yellow and w in purple.

## Data Availability

The Matlab^®^ implementations of the Madgwick and Mahoney MARG orientation estimation methods, used to generate two of the traces shown in Figure 6, are available at https://x-io.co.uk/downloads/madgwick_algorithm_matlab.zip, accessed on 16 January 2024.

## Appendix A. Specifications of the 3-Space Micro USB MARG Module from Yost Labs

Below, we transcribe the complete set of specifications offered for the 3-Space Micro USB Attitude and Heading Reference System—AHRS—MARG module from the manufacturer (Yost Labs) at https://yostlabs.com/product/3-space-micro-usb/, accessed on 3 April 2024 ([Specifications] Tab).

**Table A1:** General specifications of the 3-Space Micro USB MARG.

Parameter	Specification
Part number	TSS-MUSB (8G Accelerometer)
	TSS-MUSB-HH (24G Accelerometer)
	TSS-MUSB-H3 (400G Accelerometer)
Dimensions	23 mm × 23 mm × 2.2 mm (0.9 × 0.9 × 0.086 in.)
Weight	1.3 g (0.0458 oz)
Supply voltage	+3.3 v ~ +6.0 v
Power consumption	45 mA @ 5 v
Communication interfaces	USB 2.0, asynchronous serial
Filter update rate	up to 250 Hz with Kalman AHRS (higher with oversampling)
	up to 850 Hz with QCOMP AHRS (higher with oversampling)
	up to 1000 Hz in IMU mode
Orientation output	absolute and relative quaternion, Euler angles, axis angle, rotation matrix, two vector
Other output	raw sensor data, normalized sensor data, calibrated sensor data, temperature
Serial baud rate	1200~921,600 selectable, default: 115,200
Shock survivability	5000 g
Temperature range	−40~85 °C (−40~185 F)

**Table A2:** Specifications of the sensors (module, accelerometer, gyroscope, compass).

Parameter	Specification
Orientation range	360° about all axes
Orientation accuracy	±1° for dynamic conditions and all orientations
Orientation resolution	<0.08°
Orientation repeatability	0.085° for all orientations
Accelerometer scale	±2 g/±4 g/±8 g selectable for standard models
	±6 g/±12 g/±24 g selectable for HH models
	±100 g/±200 g/±400 g selectable for H3 models
Accelerometer resolution	14 bit, 12 bit (HH), 12 bit (H3)
Accelerometer noise density	99 µg/√Hz, 650 µg/√Hz (HH), 15 mg/√Hz (H3)
Accelerometer sensitivity	0.00024 g/digit–0.00096 g/digit
	0.003 g/digit–0.012/digit (HH)
	0.049 g/digit–0.195 g/digit (H3)
Accelerometer temperature sensitivity	±0.008%/°C, ±0.01%/°C (HH, H3)
Gyro scale	±250/±500/±1000/±2000°/s selectable
Gyro resolution	16 bit
Gyro noise density	0.009°/s/√Hz
Gyro bias stability @ 25 °C	2.5°/h average for all axes
Gyro sensitivity	0.00833°/s/digit for ±250°/s
	0.06667°/s/digit for ±2000°/s
Gyro non-linearity	0.2% full-scale
Gyro temperature sensitivity	±0.03%/°C
Compass scale	±0.88 Ga to ±8.1 Ga selectable (±1.3 Ga default)
Compass resolution	12 bit
Compass sensitivity	0.73 mGa/digit
Compass non-linearity	0.1% full-scale

## Appendix B. Pseudo-Code for Computation of Trustworthiness Parameters and Sensitivity Analysis

### Appendix B.1. Pseudocode for Computation of the Accelerometer Trustworthiness Parameter Alpha

The goal is to produce a value of alpha (0 < alpha < 1) that represents how much we can trust in accelerometer-based corrections to the MARG orientation estimate (0% to 100%).

At every sampling instant (i.e., when new MARG values are retrieved):

1. Set value of SlopeAlpha (Suggested 1.0)
2. Read (called “Confidence Factor” in the 3-Space module) from MARG
3. alpha1 = (Stillness SlopeAlpha × Stillness) + 1 − SlopeAlpha
4. alpha = [alpha1 + abs(alpha1)]/2

NOTES: Stillness, SlopeAlpha, alpha1, and alpha are all scalar variables.

### Appendix B.2. Pseudocode for Computation of the Magnetometer Trustworthiness Parameter MUK

The goal is to produce a value of MUK (0 < MUK < 1) that represents how much we can trust in accelerometer-based corrections to the MARG orientation estimate (0% to 100%).

At every sampling instant (i.e., when new MARG values have been retrieved and the final quaternion orientation estimate of the previous iteration, qOUT, is available):

1. Set value of SlopeMuk (Suggested 1.5)
2. Read current magnetometer values, m0, from MARG
3. Compute the mapping of current magnetometer readings, m0, to the inertial frame:
  m0i = qOUT ⊗ m0 ⊗ qOUT*
4. Compute the cosine of the angle lambda between moi (obtained in 3.) and the initial magnetometer reading, at startup, Minit:
  magnitude_m0i = norm(m0i) magnitude_Minit = norm(Minit) cos_lambda = dot(m0i, Minit)/(magnitude_m0i × magnitude_Minit)
5. lambda = acos(cos_lambda)
6. MuKA = 1 − (SlopeMuk × lambda)
7. penalty_MuKM = (magnitude_moi/magnitude_Minit) × lambda
8. MuKM = 1 − penalty_MuKM
9. Mu1 = [MuKA + MukM)/2
10. Mu2 = min(current and past Mu1 values in the last 0.4 s)
11. MUK = Mu2 × alpha

NOTES:

SlopeMuk, magnitude_m0i, magnitude_Minit, cos_lambda, lambda, MuKA, penalty_MuKM, MuKM, Mu1, Mu2, and MUK are all scalar variables.
m0, m0i, and Minit are 3D vectors. qOUT and its conjugate qOUT* are quaternions.
⊗ represents quaternion product; norm( ) is the magnitude of a 3D vector; dot( ) is the dot product of two 3D vectors; abs( ) is the absolute value of a scalar; × is the product of scalars.

### Appendix B.3. Sensitivity of the GMVDK Performance to Values of SlopeAlpha and SlopeMuk

The pseudocode listings shown in the previous two subsections indicate that computation of alpha and MUK requires assigning numerical values to the parameters SlopeAlpha and SlopeMuk, respectively. Equation (20) in the body of the article indicates that we used the value SlopeMuk = 1.5 in our implementations. Similarly, in calculating alpha, we used a numerical value of 1.0 for the parameter SlopeAlpha, referenced in Appendix B.1. In both cases, a larger value of the slope parameter would cause the final alpha and MUK values to drop more significantly with a given level of departure from the corresponding ideal conditions (complete absence of movement, for alpha, and complete absence of magnetic distortion, for MUK). In other words, larger SlopeAlpha or SlopeMuk parameters would cause increased caution in accepting orientation corrections based on the corresponding variable (alpha or MUK).

While we set the recommended values, SlopeAlpha = 1.0 and SlopeMuk = 1.5, on the basis of observing the performance of GMVDK in multiple recordings, it is of interest to explore how “sensitive” the performance of GMVDK might be to small shifts from the recommended values for these parameters. We sought to assess these sensitivities (interpreted as the ratio of percentual change in the GMVDK performance to the percentual change in the corresponding parameter) by empirical means, studying a representative case, which is, in fact, the one presented in Figure 6 and Figure 7 of the article. A modified version of Figure 6 is shown below (Figure A1) for the description of our sensitivity assessment procedure.

Since we are now studying the sensitivity of GMVDK to parameter changes, exclusively, it was important to establish a performance metric that involved GMVDK quaternions only. It was decided to compare the GMVDK quaternions to “ideal” reference quaternions which are known for the time intervals where Poses 1, 2, 3, 4, 5 6, 7, 8, 9, and 10 could be considered to be held by the experimental subject. Segments of the same duration were considered for each one of the poses. Therefore, the QAD values between the quaternion obtained from GMDVK and the corresponding reference quaternion for segments Seg1 to Seg10 were concatenated together into a timeseries All_Segments. Then, the RMSE based on the concatenated time series was computed. This performance value RMSE_from_GMVDK will be a larger positive number when the performance of GMVDK deteriorates, such that the quaternion obtained from GMVDK in the segments studied is more different from the reference quaternions.

Table A3 shows (in its bottom row) the RMSE_from_GMVDK measured as the parameter SlopeAlpha was assigned three values that are lower than 1.0, then it was assigned the recommended value of 1.0 (bolded column), and then three values that were higher than 1.0. The difference in the SlopeAlpha values of any two adjacent columns was 0.25 (25% of the “nominal” value SlopeAlpha = 1.0). All along the value of the other parameter, SlopeMuk was held constant at the “nominal” value of 1.5.

**Table A3:** RMSE of the orientation quaternion from GMVDK (SlopeMuk held at 1.5).

SlopeAlpha	0.25	0.50	0.75	**1.00**	1.25	1.50	1.75
RMSE_from_GMVDK (°)	9.451	9.821	9.995	**9.843**	9.574	9.525	9.566

The bolded column contains the nominal parameter and resulting RMSE. This column and the two adjacent ones (filled in color) are further explored in the next table.

We observed that RMSE_from_GMVDK did not change much for any of the six non-nominal values tested. Table A4 now shows the values of just the shaded columns from Table A3 normalized by the corresponding nominal value, in order to express them as percentual changes:

**Table A4:** RMSE_from_GMVDK (%) (SlopeMuk held at 1.5).

SlopeAlpha	75%	**100%**	125%
RMSE_from_GMVDK	101.55%	**100%**	97.27%

The empirical assessment of the sensitivity of the algorithm’s performance to variations in this parameter may be approximated as the average of the one-step forward differences before and after the nominal assignment of SlopeAlpha = 1.0, using the percentual values from Table A4:

(A1) $$SlopeAlpha_{sensitivity}=\frac{\left[\left(100-101.55\right)+(97.27-100)\right]/2}{\left[\left(100-75\right)+(125-100)\right]/2}=\frac{-2.14\%}{25\%}=-0.0856$$

The sensitivity of GMVDK performance with respect to changes in the value used for the parameter SlopeMuk was investigated in the same way. Table A5 shows (in its bottom row) the RMSE_from_GMVDK measured as the parameter SlopeMuk was assigned three values that were lower than 1.5, then it was assigned the recommended value of 1.5 (bolded column), and then three values that were higher than 1.5. The difference in the SlopeMuk values of any two adjacent columns was 0.25 (16.67% of the “nominal” value SlopeMuk = 1.5). All along the value of the other parameter, SlopeAlpha was held constant at the “nominal” value of 1.0.

**Table A5:** RMSE of the orientation quaternion from GMVDK (SlopeAlpha held at 1.0).

SlopeMuk	0.75	1.00	1.25	**1.50**	1.75	2.00	2.25
RMSE_from_GMVDK (°)	18.691	10.523	10.059	**9.843**	9.730	9.686	9.654

The bolded column contains the nominal parameter and resulting RMSE. This column and the two adjacent ones (filled in color) are further explored in the next table.

In this case, we observed that the performance of GMVDK remained stable when the value of SlopeMuk was in the neighborhood of the nominal value (e.g., 1.25 < SlopeMuk < 1.75, or even 1.00 < SlopeMuk < 2.00), but if SlopeMuk was decreased too much (e.g., assigning SlopeMuk = 0.75, instead of the suggested SlopeMuk = 1.5), the algorithm did not penalize the presence of magnetic disturbances enough, allowing inappropriate magnetometer-based corrections, which degraded the performance of the algorithm noticeably.

We approximated the sensitivity to changes of SlopeMuk in the neighborhood of the nominal value SlopeMuk = 1.5 with the same procedure as that used for estimating the sensitivity to changes in SlopeAlpha. Table A6 shows the percentual changes:

**Table A6:** RMSE_from_GMVDK (%) (SlopeAlpha held at 1.0).

SlopeMuk	83.33%	**100%**	116.66%
RMSE_from_GMVDK	102.20%	**100%**	98.86%

Then, proceeding with the calculation of the sensitivity:

(A2) $${SlopeMuk}_{sensitivity}=\frac{\left[\left(100-102.20\right)+(98.86-100)\right]/2}{\left[\left(100-83.33\right)+(116.66-100)\right]/2}=\frac{-1.67\%}{16.66\%}=-0.1002$$

Overall, the estimated values of these sensitivities (−0.0856 and −0.1002) seemed to indicate that small shifts from the nominal values assigned to both these parameters may not have had a large detrimental effect in the performance of the algorithm. The numbers in Table A3 and Table A5 seem to indicate that a large change of the nominal SlopeMuk = 1.5 value (for example to a value of 0.75) will a have noticeable negative impact in the performance of the algorithm.

## Appendix C. GMVDK Performance at Varying Levels and Directions of Magnetic Disruption

In order to evaluate the performance of GMVDK (and the onboard implementation of the Kalman filter included by the manufacturer of the 3-Space Micro USB MARG), we analyzed recordings of two alternative setups:

### Appendix C.1. Magnetic Disruptor Not Placed at Location B, but 15 cm South from Location B

While the trajectory and sequence of rotations applied to the MARG remained the same as for all the experiments in the body of the article (summarized in Table 2), placing the magnetic disruptor 15 cm to the south of Location B, where the MARG was made to hold Poses 6 to 10, implies that the magnetic disruption challenging the algorithm while these poses were held was different, possibly lower (since there was a longer distance to the disrupter). Figure A2 shows the plot of the orientation quaternions obtained from GMVDK and from the onboard Kalman filter.

The similarity of the quaternions obtained for the second set of five poses to the quaternions obtained for the first set of five poses was clearly greater for the output from GMVDK, indicating that it was still more resilient to the magnetic disruption than the Kalman filter. Further, the RMSE values of QAD before and after the vertical dividing line (shown inside the lower panel of the figure) confirmed that the Kalman filter output became more different from the output of GMVDK in the second half of the recording, although in this case, as the magnetic disrupter was farther away, the difference is smaller than the corresponding difference shown in Figure 4 (in the body of the manuscript).

### Appendix C.2. Magnetic Disruptor Not Placed at Location B, but 15 cm North from Location B

In this second additional experiment, the trajectory and sequence of rotations applied to the MARG remained the same as for all the experiments in the body of the article (summarized in Table 2), but the magnetic disruptor was placed 15 cm to the north of Location B, where the MARG was made to hold Positions 6 to 10. This alternative positioning of the disruptor also implies that the magnetic disruption challenging the algorithm while these poses were held was different, possibly lower (since there was a longer distance to the disrupter). It is worth noting that the alternative position of the magnetic distortion will modify the lines of magnetic field at Location B in yet a third different way. Figure A3 shows the plot of the orientation quaternions obtained from GMVDK and from the onboard Kalman filter.

Interestingly, the Kalman filter in this case seemed to yield orientation estimates that were less affected during the second half of the recording. As in previous cases, the orientation estimates yielded by GMVDK during the second half of the recording are very similar to the ones it yielded for the same 5 poses during the first half of the recording. It seems, therefore, that the level of deterioration of the Kalman filter estimates did not only depend on the closeness of the MARG to the magnetic disruptor but also on the specific directions taken by the lines of magnetic field where the MARG was. The lower level of deterioration of the Kalman filter estimates was confirmed by the similarity of RMSE levels of QAD obtained for the first and the second halves of the recording.

In any case, the results of these two additional experiments seem to indicate that GMVDK displays a significance level of resilience to magnetic distortion over a range of magnetic abnormality levels.

## Appendix D. H-B-A-H Route Testing

We sought to observe if GMVDK has the ability to “recover” from disruptions suffered in its computation by magnetic irregularities once the MARG is translated away from the magnetically distorted area. We were also interested in comparing this kind of ability as it may exist in GMVDK and in other compensation methods (e.g., Kalman filter). Therefore, we present here the results of applying GMVDK and the onboard Kalman filter algorithm to a recording in which the MARG was translated from the home location (H) to the magnetically disturbed Location B first, where the five poses were held. Only then was the MARG translated to the location that did not have a magnetic disruptor under it (Location A). After holding the same five poses in Location A, the MARG was translated back to the home location (H), and the recording ended. Figure A4 shows the quaternions yielded by GMVDK and the Kalman filter, as well as the quaternion angle difference (QAD) between the two series of quaternions, indicating the RMSE QAD values for the first and second halves of the recording, defined by the vertical dividing line in the plots.

Figure A4 shows that the performance of the Kalman filter was significantly deteriorated after translating the MARG from Location H to Location B, yielding a large RMSE QAD value of 145.72° for the first half of the recording. The disruption for GMVDK was much smaller and disappeared very shortly after the vertical line, whereas the Kalman filter took 2 or 3 milliseconds to “recover” until it provided orientation results that were similar to those provided by GMVDK, so that the RMSE QAD of the second half of the recording was much lower. Interestingly, after coming out of the magnetically disrupted Location B, the quaternions generated by the Kalman filter were close to the quaternion generated by GMVDK but with all four of its components multiplied times “−1”. In the context of quaternions, one quaternion and another one that was the same as the first with all its four components multiplied by “−1” indicated the same orientation.
